# Overview of Recent Developments in Composite Epoxy Resin in Organic Coating on Steel (2020–2024)

**DOI:** 10.3390/ma18071531

**Published:** 2025-03-28

**Authors:** Jianghua Hao, Kun Yang, Jiaye Wu, Mingzhu Wu, Ying Li

**Affiliations:** 1 Chemical Pollution Control Chongqing Applied Technology Extension Center of Higher Vocational Colleges, Chongqing Industry Polytechnic College, Chongqing 401120, China; haojianghua6625@163.com (J.H.); hahayangkun@163.com (K.Y.); wujy@cqipc.edu.cn (J.W.); 2 Avic Xi’an Aircraft Industry Group Co., Ltd., Xi’an 710089, China; 3 Avic Shenyang Aircraft Corporation, Shenyang 110086, China

**Keywords:** epoxy coating, modification, steel, anti-corrosion performance

## Abstract

Epoxy resin, widely recognized for its excellent performance, is extensively applied in the anti-corrosion field of steel. Continuous enhancement of the anti-corrosion performance of epoxy resins to satisfy more stringent requirements has become a current hot topic of interest in both scientific and industrial circles. This review focuses on recent advancements in composite epoxy resin coatings for steel from 2020 to 2024, emphasizing improvements in anti-corrosion performance through various additive modifications. Modification methods are categorized into metal-based compounds, organic compounds, organometallic compounds, and carbon-based materials. To assist scholars in understanding the latest research advancements, key findings from electrochemical tests, mechanical assessments, and structural characterizations are summarized, highlighting their influence on corrosion resistance, adhesion, mechanical properties, and self-healing capabilities.

## 1. Introduction

As a popular structural material, steel is frequently exposed to aggressive environments that accelerate its corrosion. High humidity is one such condition, where water vapor can condense on the steel surface, triggering electrochemical corrosion reactions. Additionally, steel structures located near coastal areas or in chemical industrial zones are continuously subjected to salt spray or corrosive chemicals, further intensifying the corrosion process. The consequences of steel corrosion are twofold: it weakens the structural integrity, and it leads to significant economic losses from repair and replacement costs.

The use of organic coatings to protect metals stands as one of the most prevalent anti-corrosion strategies. These coatings act as a shield, effectively isolating the metal from its surrounding environment and thus preventing damage to the substrate [1]. Epoxy resins, recognized as top-tier anti-corrosion materials, are widely used to protect metals in industry. This is largely attributable to their exceptional mechanical robustness, thermal stability, and chemical resistance [2]. Traditional epoxy coatings have been widely used for their good mechanical properties and corrosion resistance. However, recent modifications have significantly enhanced their performance. For example, metal-based compounds like ZnO and TiO_2_ improve barrier properties and active corrosion protection. Organic compounds such as polyaniline enhance adhesion and self-healing capabilities. Organometallic compounds like MOFs offer tunable structures for improved corrosion resistance, while carbon-based materials like graphene provide excellent barrier properties and mechanical strength. These modifications address the limitations of traditional epoxy coatings, offering superior protection in harsh environments. To continuously improve their performance under various application environments, a variety of methods and numerous novel composites have been employed to engineer the structure of epoxy coating.

In this review, we summarize selected typical examples of composite epoxy resin coatings for steel, drawn from peer-reviewed scientific papers from 2020 to 2024. According to the characteristics of the modified coatings, the additives are classified into four categories for in-depth analysis and discussion. These categories are metals and their compounds, organic compounds, organometallic compounds, and carbon materials. Within the metals and their compounds category, inorganic/organic hybrid complexes and silicon compounds are included in this review. It should be noted, however, that an unambiguous classification of certain substances can be quite challenging. Moreover, our discussion does not encompass activation mechanisms and specific testing methodologies.

## 2. Epoxy Coatings Modified by Metals and Their Compounds

The anti-corrosion performance of epoxy resins modified by metals and their compounds, particularly metal oxides, has been significantly enhanced [3,4]. A wide variety of metal oxides and modified metal oxides, such as ZnO [5], TiO_2_ [6], CeO_2_ [7], NiO [8], Fe_3_O_4_ [9], among others, have been added into epoxy resins to optimize the properties of organic coatings on the surface of steel. Through an analysis of XRD, FT-IR, TEM, XPS, and EIS, Kumari et al. [4] revealed that metal oxide (ZnO, TiO_2_) and graphene oxide could enhance the interaction of epoxy resin film and the metal surface via the formation of metal–oxygen bonds. This enhanced interaction provided improved corrosion protection for steel in a harsh environment.

### 2.1. Modification of Zinc-Rich Epoxy Coating

**Figure 1:**
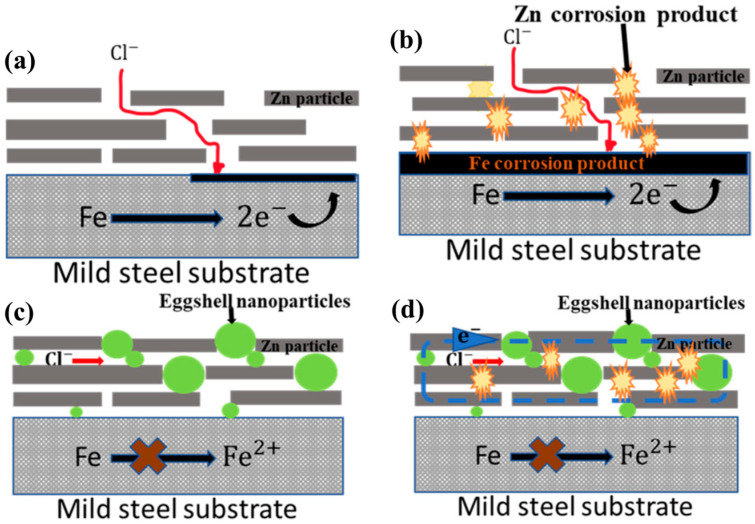
Enhancement mechanism of epoxy coating modified by eggshell nanoparticles ((**a**) ZREC with interstitial spaces for permeation of chloride species; (**b**) ZREC coated mild steel with zinc corrosion products; (**c**) ZENE-5-C coating showing treated eggshell nanoparticles within the interstitial spaces of zinc particles at the start of immersion; (**d**) ZENE-5-C coating showing electrical contact between zincparticles and treated eggshell nanoparticles with deposition of zinc corrosion products after 30 days immersion). [14].

Among metals and their compounds, zinc-rich organic coatings have been successfully applied since the 1930s because of their excellent resistance to chemical corrosion [10,11]. Open-circuit potential (OP) and electrochemical impedance spectroscopy (EIS) tests showed that a zinc-rich organic coating (80 wt% zinc particles) provided better cathodic protection for carbon steel [12]. Pandis et al. [10] found that commercial zinc-rich organic coatings (ZN691) could effectively prevent 304 L stainless steel from being corroded under highly acidic media (40% H_2_SO_4_, 40% HNO_3_, and 37% HCl). The authors proved that the anti-corrosion performance of two commercial zinc-rich organic coatings (ZN691 and ANORZINC) was higher than that of an FeO-rich organic coating (TROPIS PRIMER CL). The anti-corrosion performance of zinc-rich organic coatings was remarkably improved when 0.5 wt% zinc fibers were added to the mixture system [13]. Due to the high calcite content, eggshell wastes were studied as additives to enhance the corrosion resistance properties of zinc-rich coatings [14]. Electrochemical analysis revealed that added eggshell wastes could enhance the anti-corrosion properties of zinc-rich epoxy coatings because the chloride ions were successfully prevented by eggshell nanoparticles (NPs) from contacting the substrate surface (Figure 1). Qi et al. [11] investigated the corrosion protection performances of zinc-rich coatings when spherical zinc particles were partially replaced by stainless steel flakes, and they considered the enhancement to be attributed to the synergistic effects of zinc and steel. Haddadi et al. [15] synthesized Zn-FALE (zinc cations/*Ferula Asafoetida* leaves extract) and revealed that the material significantly enhanced the barrier and active corrosion protection performance of epoxy coatings. Galvanized steel showed outstanding adhesion strength and corrosion resistance once its surface was treated by Zn-Al layered double hydroxide (LDH) grown in situ [16]. Rahmani et al. [17] used zinc cation and 3-nitrobenzoic acid as raw materials to treat carbon steel by a pulse-reverse electrodeposition method. The treated carbon steel was then laid over epoxy resin and showed stronger adhesion and anti-corrosion performance. Compared with the anti-corrosion performance and mechanical properties of epoxy coatings modified with nano hybrid ZnO particles, Kabaoglu et al. [18] found that a micro ZnO/Epoxy composite coating on steel plate had superior barrier protection. Superhydrophobic coating technology was also utilized to treat carbon steel, and the experimental results showed that a nano zinc oxide/epoxy coating significantly improved the corrosion protection of the substrate [5]. To enhance anti-corrosion performance, organotitanate-modified ZnO NPs were prepared and tested [19]. As the modified ZnO NPs could be dispersed in epoxy resin better than the unmodified ZnO NPs, the modified ZnO NPs improved the mechanical properties, chemical resistance, thermal stability, and anti-corrosion protection ability of epoxy resin coating [19]. Xavier [3] synthesized 3-amino-1,2,4-triazole-5-thiol-functionalized Al_2_O_3_-ZnO NPs, and the author found that epoxy resin with added Al_2_O_3_-ZnO NPs had excellent barrier strength and mechanical properties. Moreover, ZnO NPs were added to an organic–inorganic hybrid coating fabricated by bisphenol A diglycidyl ether epoxy resin with silica NPs [20]. The obtained dual-function coating showed higher anti-corrosion and photocatalysis activities. Nano ZnO and micro ZnO have different effects on epoxy coatings. Nano ZnO provides better dispersion and a higher surface area-to-volume ratio, enhancing corrosion protection through improved barrier properties and active inhibition. Micro ZnO, on the other hand, offers superior mechanical properties and easier processing. The choice between nano ZnO and micro ZnO depends on the specific application requirements, with nano ZnO being more suitable for high-corrosion environments and micro ZnO being more suitable for applications where mechanical strength is crucial.

### 2.2. Titanium-Modified Epoxy Coatings

Titanium compounds as additives in epoxy coatings have also been extensively studied for the anti-corrosion protection of steel [6,21,22,23,24,25,26]. Dagdag et al. [21] found that the anti-corrosion capability of carbon steel was enhanced in 3% NaCl solution once epoxy resin and TiO_2_ composite, serving as anti-corrosive materials, were coated. The authors explained that the fundamental anti-corrosion mechanism was TiO_2_ blocking the surface micropores and hindering the penetration or diffusion of corrosive species. To future improve the corrosion protection, TiO_2_ NPs were fabricated with poly-dimethylamino siloxane and applied to steel petroleum tanker trucks. The painted tanker displayed excellent anti-corrosion, mechanical, chemical, electrical, thermal, and UV resistance [22]. As a starting material, TiO_2_ coupled with silane was added into epoxy coating to boost the corrosion resistance of electrical steel [26]. Compared to the conventional phosphate coating, the silane-modified TiO_2_ had better dispersion stability [26]. In addition, a mixture of TiO_2_ and graphite was added into epoxy resin to enhance the thermal resistance of the coating [27].

**Figure 2:**
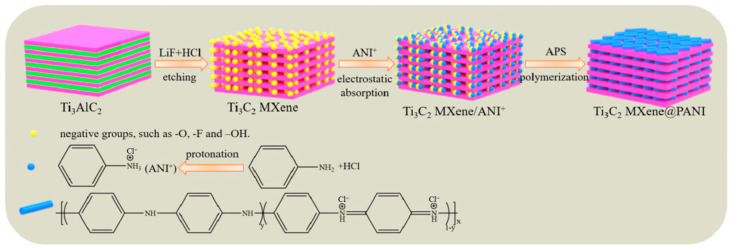
Synthesis of Ti_3_C_2_ MXene@PANI composites by etching Al layer of the precursor Ti_3_AlC_2_ using LiF and HCl treatment [25].

Recently, MXene polymer hybrid materials have attracted significant attention due to their outstanding resistance to permeation, diverse surface chemical properties, impressive mechanical properties, and metal-like electrical and thermal conductivity [28]. As a kind of MXene polymer hybrid material, epoxy resins with Ti_3_C_2_T_x_ MXene were also investigated as an additive for high-performance organic coatings for steel. L-cysteine-modified Ti_3_C_2_T_x_ MXene nanosheets were fabricated by Li’s group [23] to improve the anti-corrosion performance of waterborne epoxy coatings. The results indicated that a good dispersion of Ti_3_C_2_T_x_ MXene enhanced the corrosion resistance of epoxy resin coatings. Due to their excellent barrier performance and mechanical properties, Ti_3_C_2_T_x_ (x = 0) MXene nanosheets were synthesized and thoroughly studied as additives in epoxy coatings (Figure 2) [25]. Experiments revealed that the synthesized Ti_3_C_2_ MXene@PANI (polyaniline) composites could increase the corrosion resistance by 1–2 orders of magnitude compared to pure waterborne epoxy coating in a 3.5 wt% NaCl solution. By a hierarchical design method, Zhang et al. [29] demonstrated that the thermal, tribological, and anti-corrosive performances of steel were significantly enhanced by a microcapsule-based epoxy coating modified with Ti_3_C_2_T_x_. Moreover, Ti_3_C_2_T_x_ MXenes, in combination with other treatment techniques, were applied to strengthen the anti-corrosion of other metal alloys [24].

### 2.3. Silicon-Modified Epoxy Coatings

**Figure 3:**
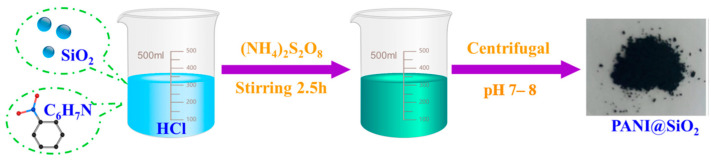
Synthesis of the core–shell structural PANI@SiO_2_ [31].

**Figure 4:**
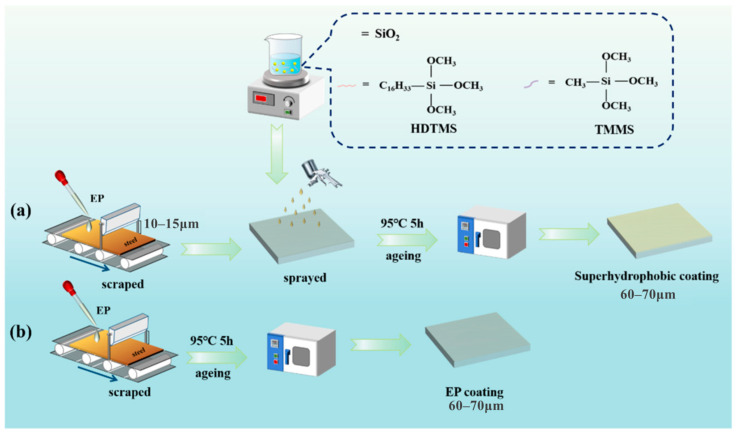
Preparation of superhydrophobic HDTMS@SiO_2_ coating and EP coating on the Q235 carbon steel ((**a**) the superhydrophobic HDTMS@SiO2 coating and (**b**) the EP coating on the Q235 carbon steel substrate) [39].

Given that silicon belongs to the metal-like elements, we classify silicon compounds as a type of metal–organic coating in this review. As effective organic coating modification reagents, organic silanes are widely used for doping epoxy coatings on steel. On the carbon steel surface, a multilayer silane-doped epoxy coating exhibited remarkable anti-corrosion and adhesion performance [30]. To enhance the corrosion resistance of steel fibers, core–shell structural PANI@SiO_2_ was synthesized via an in situ polymerization method (Figure 3), and a 2 wt% PANI@SiO_2_/epoxy coating showed the most excellent resistance to stress corrosion and electrochemical corrosion [31]. To reduce the delamination rate of the coating from the matrix, Fernández-Alvarez et al. [32] modified SiO_2_ with calcium using an ion-exchange method for incorporation into organic coatings. The experimental data confirmed that this treatment method could strengthen the scratch resistance, universal hardness, and wear resistance. On the surface of steel, Fe-O-M (metal) and/or Fe-N-M bonds effectively resist steel corrosion. Atta et al. [33] investigated the performance of epoxy coatings separately hybridized with amino-functionalized calcined silica NPs and NIPAM-VTS-polysiloxane (N-isopropylacrylamide-co-vinyltrimethoxysilane). The amino-functionalized hybrid silica epoxy coatings were found to be superior in terms of adhesion, mechanical testing, and corrosion protection over those of hybrid materials without amino-functionalization. The authors also demonstrated that the amino-functionalized NIPAM-VTS-polysiloxane exhibited novel self-healing performance [34]. All data indicated that Fe-O-Si and Fe-N-Si bonds strengthened the adhesion strength of epoxy coating, resulting in enhanced anti-corrosion performance. Hybrid silica NPs were prepared with silica and 2-chlorothioxanthone under mild preparation conditions [35]. The synthesized hybrid NPs displayed higher photopolymerized efficiency and dispersion properties than pure 2-chlorothioxanthone, indicating that the coating had excellent UV-curing performance. Silica hybridized with chitosan was induced into epoxy resin to promote the active barrier effect on the metal surface. The data from FT-IR, UV–vis, and EIS analyses showed that the active barrier effect on the metal surface had significant anticorrosive activity. 3-(triethoxysilyl)propyl isocyanate was grafted onto silica surface modified by 1-(3′-aminopropyl) imidazole to prepare nanofillers [36]. The engineered nanofillers were then dispersed into epoxy resin to obtain epoxy nanocomposite coatings. The experimental results indicated that both the mechanical strength and corrosion resistance were improved. Through pre-electrodeposited silane treatment, the interaction of epoxy coating and steel was enhanced, indicating that the corrosion resistance was reinforced [37]. Zhu et al. [38] fabricated triazine-modified silica and significantly enhanced the anti-corrosion property of epoxy coatings on carbon steel. Zeng et al. [39] tested the anti-corrosion properties of durable superhydrophobic silica/epoxy resin coating applied to steel. The results clearly showed that the substrate was well protected in high-salt (3.5% NaCl solution) and high-H_2_S environments (Figure 4). Superhydrophobic mesoporous silica (MCM-41) was added to epoxy coatings to alter the wettability and anti-corrosion behavior of the matrix surface [40]. The Al_2_O_3_-SiO_2_ in MCM-41 was fabricated with poly(o-toluidine) and incorporated into epoxy resin. The modified resin NPs were painted onto carbon steel, and the corrosion tests showed that the Al_2_O_3_-SiO_2_ improved the protective features of the coating. Sol–gel-based silicon coatings have gained attention for their excellent corrosion resistance and mechanical properties. So, sol–gel technology was employed to produce organic–inorganic coatings containing silica [41]. The investigations showed that the coating prepared using the sol–gel method exhibited good endurance capacity under high-temperature and salt spray conditions due to the formation of highly cross-linked networks, enhancing barrier properties and chemical resistance. These sol-gel coatings also exhibit good adhesion to steel surfaces and can be tailored to specific applications through the incorporation of different silicon compounds. Their advantages include improved thermal stability, UV resistance, and durability in harsh environments, making them a promising alternative to traditional epoxy coatings. To improve the solubility of zinc phosphate in epoxy resin, three mixtures of NaA zeolite and zinc monophosphate (1:1; 3:1 and 1:3) were studied [42]. EIS tests found that the epoxy coating on low-alloy 09G2S carbon steel had the highest corrosion resistance when the ratio of NaA zeolite to zinc monophosphate was 1:3.

As additives, natural minerals containing silicon were also investigated in epoxy coatings [43,44,45]. For example, kaolin mixed with graphite as an additive was utilized to enhance the thermal stability of epoxy coating on a steel surface [27]. The test results indicated that the substrate exhibited greater thermal resistance due to the formation of a homogeneous organic–ceramic coating. In another study, hydrophobic montmorillonite (HMT) clay was modified with silver and iron oxides to produce hybrid NPs [46]. When these modified HMT NPs were blended with epoxy resin, the resulting coating demonstrated extraordinary adhesion, mechanical strength, chemical resistance, and abrasion resistance under a high-salinity environment. Additionally, Beryl and Xavier [47] studied the electrochemical and mechanical properties of silanized clay-epoxy coatings on mild steel. All the test data revealed that the modified clay was effectively dispersed within the epoxy resin, leading to a dramatic improvement in the coating’s anti-corrosion performance. The same research group also investigated the barrier, hydrophobic, and mechanical properties of epoxy coating mixed with silanized halloysite NPs on steel exposed to natural seawater [45]. The authors claimed that diethoxy(3-glycidyloxypropyl) methylsilane-modified clay epoxy coating exhibited stronger adhesive properties and a lower corrosion rate (0.0017 mm/year). Furthermore, the UV stability of epoxy coating was improved by incorporating halloysite nanotubes, encapsulated with organic UV stabilizers and lignin [44]. The epoxy system containing 2 wt% of encapsulated halloysite showed significantly higher UV stability compared to pure epoxy resin. In terms of other additives, phenyl-modified etched basalt was fabricated into an epoxy resin layer to improve the anti-corrosion performance of coatings [48]. The modified basalt constructed micro–nano capillary structures and generated π electrons, which contributed to the enhanced performance. Lastly, organically fabricated sepiolite nanofillers were added to epoxy resins to boost the anti-corrosion resistance of surface coatings for mild carbon steel [43].

### 2.4. Epoxy Coatings Modified by Other Metal Compounds

**Figure 5:**
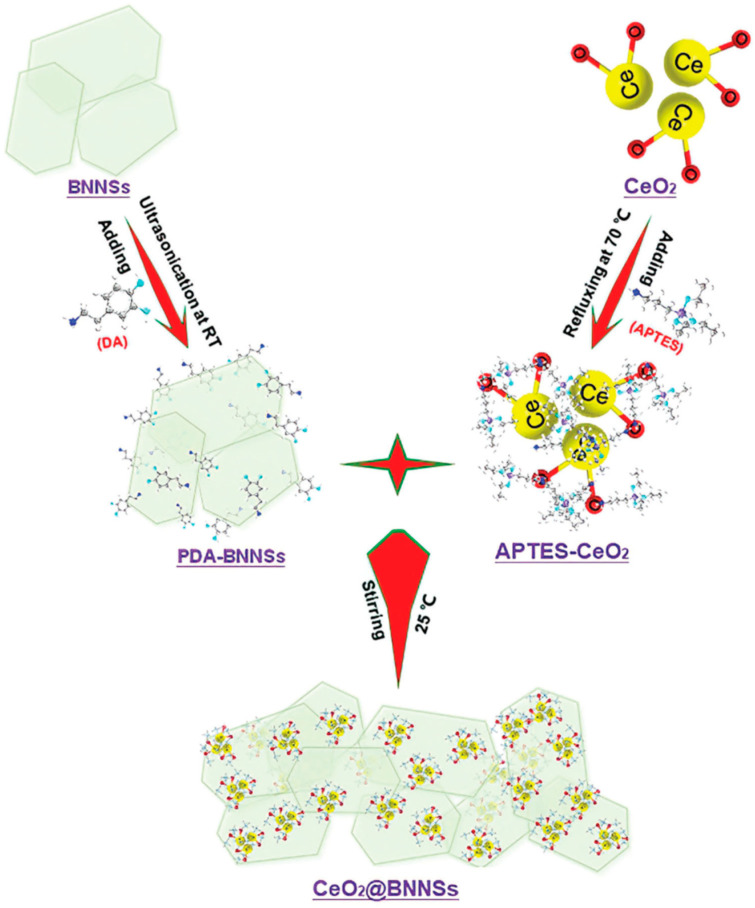
Synthesis route of CeO_2_@BNNSs [50].

Due to their low costs, ease of synthesis, and chemical stability, cerium oxide (CeO_2_) and polyaniline (PANI) have been incorporated into epoxy resin to enhance the anti-corrosive properties of organic coatings [7,49]. Fan et al. [49] prepared a CeO_2_-PANI conductive polymer via in situ chemical oxidation polymerization. The prepared polymer was dispersed in F51 epoxy resin and sprayed onto a steel surface to boost its corrosion resistance. The authors highlighted that the synergistic interaction between PANI and Ce^3+^ ions was crucial for enhancing the steel’s anti-corrosion properties. Similarly, Lei et al. [7] demonstrated that PANI/CeO_2_ NPs exhibited excellent anti-corrosive performance for epoxy coating on carbon steel in 3.5 wt% NaCl solution. They attributed this to PANI’s redox behavior, which improved the coating’s self-healing ability. Dun et al. [50] developed CeO_2_@BNNSs (BN, boron nitride) fillers (Figure 5) and dispersed them in epoxy resin to create high-quality coatings for steel surfaces. This significantly improved the corrosion resistance in 3.5 wt% NaCl solution. Wan et al. [51] synthesized novel PDA-BN@f-Al_2_O_3_ hybrids us γ-Aminopropyltriethoxysilane (KH550), Al_2_O_3_, polydopamine (PDA), and hexagonal boron-nitride (h-BN). Compared to individual Al_2_O_3_ and h-BN, PDA-BN@f-Al_2_O_3_ enhanced the barrier properties and corrosion resistance of epoxy coatings when mixed in. Micro and nano Al_2_O_3_ particles were also added to epoxy coatings to intensify the barrier and mechanical performance of steel [52]. The results showed that the tensile strength, strain, toughness, and flexural properties, respectively, were enhanced with adding 1 wt% micro Al_2_O_3_ before 1 wt% nano Al_2_O_3_ in the coating [52]. Magnetic Fe_3_O_4_ NPs modified with oleic acid were incorporated into epoxy resin to form in situ repairable coatings. Experiments confirmed that the Fe_3_O_4_ NPs prevented micro-channel formation and enhanced coating adhesion through target-driving and competitive wetting mechanisms [9]. Inspired by “Band Aid”, this group modified epoxy binder with magnetic Fe_3_O_4_ NPs [53], showing high self-repair capability for organic coatings in seawater. Additionally, Ni-Fe LDHs partially replaced with MoO_4_^−2^ efficiently improved the corrosion resistance of carbon steel (Q235) [54]. Nano MoO_3_ treated with epoxy-silane was painted onto mild steel to elevate the anti-corrosion, adhesion, and mechanical properties [55]. After silane treatment, MoS_2_ NPs were embedded into steel epoxy coatings [56]. The experiments indicated that MoS_2_ with amino silane had better corrosion resistance than with neat epoxy silane. ZrO_2_@rGO (reduced graphene oxide) was synthesized by a hydrothermal method and mixed into epoxy resin by a solvent-mixing method to create a hybrid coating [57], exhibiting excellent wear resistance and anti-corrosion performance. Multifunctional 2-amino-5-(methylthio)-1,3,4-thiadiazole (AMTTD) was utilized to modify ZrC [58]. The hybridized AMMTTD/ZrC was added into epoxy resin to increase the hydrophobicity, mechanical strength, and electrochemical properties of the coating in seawater. The anti-corrosion performance of epoxy coatings was enhanced by treating a steel surface with nanostructured samarium oxide-poly-dopamine film [59]. For 15CDV6 steel, electrocatalytically deposited cadmium improved the anti-corrosion of macromolecular epoxy coatings in 3 wt% NaCl solution by enhancing coating adhesion [60].

Table 1 provides a concise overview of the advantages and limitations of different corrosion protection methods discussed in this review. It is designed to help readers quickly compare and contrast the various approaches, facilitating informed decision-making for specific industrial applications.

## 3. Epoxy Coatings Modified by Organic Compounds 

### 3.1. Epoxy Coatings Modified by N Organic Compounds

**Figure 6:**
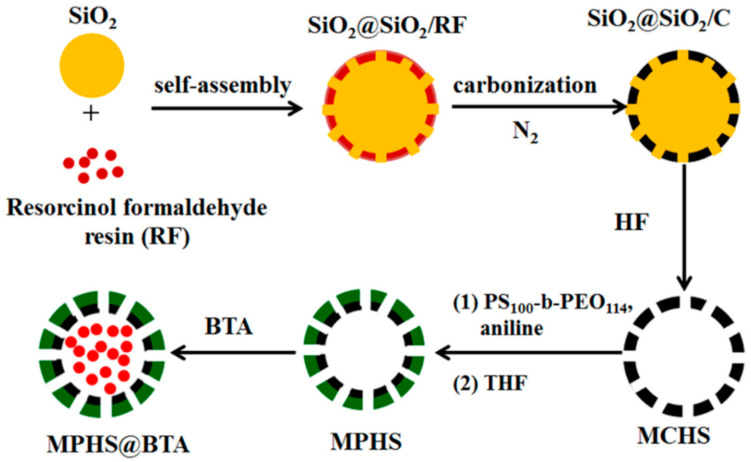
Synthesis of mesoporous polyaniline hollow spheres loaded with benzotriazole (MPHS@BTA) [62].

As modifiers, amines are widely researched in organic coatings due to their exceptional reactivity with epoxy resin. Among these amines, PANI is more commonly used to construct the structure of the epoxy coating to improve corrosion resistance. Sun et al. [61] studied how PANI filler reacted with waterborne epoxy resin to fabricate anti-corrosion coatings on a 304 stainless steel surface. The corrosion protection performance of the prepared coatings was evaluated by EIS and potentiodynamic polarization in 3.5 wt% NaCl solution, with all data revealing that the substrate was effectively protected by the coating. Due to its high corrosion inhibitor loading rate, mesoporous polyaniline hollow spheres loaded with benzotriazole were synthesized and introduced into epoxy resin to obtain self-healing coating materials (Figure 6) [62]. After a systematic investigation, the experimental results indicated that not only did the synergistic effect of the passivation function of PANI contribute to enhancing the corrosion resistance of epoxy coating, but the corrosion inhibition effect of benzotriazole also played a very helpful role. Acid-doped PANI also enhanced the anti-corrosion properties of coatings [63,64]. Yao et al. [64] found that PANI doped with 2-hydroxyphosphonocarboxylic acid could reinforce the corrosion protection performance and self-healing function of epoxy coatings. Hu et al. [63] investigated the properties of PANI doped with hydrochloric acid, *p*-toluenesulfonic acid, and dodecylbenzene sulfonic acid, respectively. Epoxy resins mixed with the doped PANIs were coated on carbon steel surfaces and evaluated by electrochemical corrosion measurements in a 3.5% NaCl solution. The results indicated that the dodecylbenzene sulfonic acid-doped PANI epoxy coating showed the best barrier effect. Liu et al. [65] researched the effects of polyaniline-based (PANP) plates added into epoxy resin and found that only 1.0 wt% PANPs could dramatically improve the corrosion protective performance of the epoxy coating. Diraki and Omanovic [66] revealed that the corrosion protection of carbon steel surfaces was enhanced once the substrate was successively painted with PANI and epoxy resin. Using *p*-aminobenzoic acid as a linker, a PANI-BN powder was synthesized using PANI and BN as raw materials [67]. Electrochemical tests displayed that the PANI-BN epoxy coating on hot-dip galvanized steel had outstanding corrosion resistance in NaCl solution. Moreover, PANI has been used to modify metal oxides to improve the anti-corrosion performance of organic coatings [7,25,31,49]. This approach leverages the unique properties of PANI to enhance the protective capabilities of metal-oxide-based coatings, offering a promising avenue for developing advanced anti-corrosive materials.

Doped PANI coatings show enhanced electrical conductivity and corrosion protection compared to undoped PANI coatings. Doping with acids or other agents improves the protonation state of PANI, leading to better charge transfer and inhibition of corrosive species. Doped PANI coatings also exhibit improved mechanical properties and adhesion to steel surfaces. The choice of dopant and doping level can be optimized to achieve the desired balance between conductivity, corrosion resistance, and processability.

**Figure 7:**
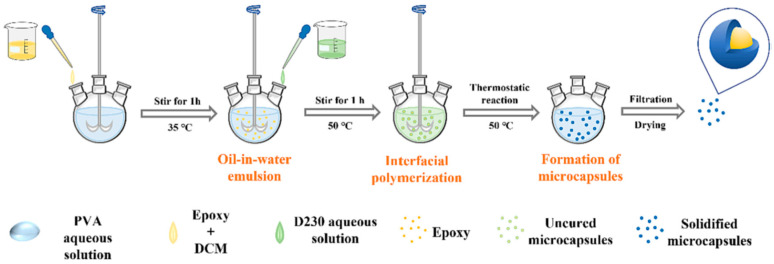
Synthesis of epoxy microcapsules [68].

Besides PANI, other nitrogen-containing organics were employed to enhance epoxy coating properties. Polyether amine D230 reacted with epoxy to form the poly(epoxy-amine) microcapsules (Figure 7), embedding liquid epoxy resin [68]. The resulting epoxy coating exhibited superior corrosion resistance due to its strong self-healing capability. Bi et al. [69] found that urea-formaldehyde could mitigate epoxy coating’s negative impact on Q235 carbon steel, improving mechanical strength and chemical stability. Curing epoxy resin with methylene dianiline enhanced its adhesion to carbon steel [70]. EIS and potentiodynamic polarization (PDP) analysis showed that methylene dianiline addition boosted the anti-corrosion properties of the coating. Liu et al. [71] synthesized the COF material LZU-1 via a reaction between *p*-phenylenediamine and pyrogallol. LZU-1 was combined with benzotriazole (BTA) to create LZU-1@BTA, which was dispersed in epoxy resin to improve its anti-corrosion properties. Tetraaniline (ATA) doped with hydrophobic polyhedral oligomeric silsesquioxane (POSS) and protonic acid was designed and synthesized, respectively [72]. EIS and salt spray tests revealed that the coating containing with 0.5 wt% POSS–ATA had the highest resistance among the tested systems. Atta et al. [73] successfully applied imidazolium cationic ionic liquids to modify the thermomechanical, toughness, and anti-corrosion characteristics of epoxy coating, as the fabricated epoxy coating’s curing networks formed more easily than commercial epoxy/amine systems. Xia et al. [74] studied the physicochemical properties of poly(arylene ether nitrile)-reinforced epoxy coating, finding that the coating’s mechanical and corrosion protection capabilities were enhanced when poly(arylene ether nitrile) was incorporated. As a carbon steel anti-corrosive coating, triglycidyl ether triethoxy triazine epoxy resin cured with 1,6-diaminohexane and 4,4-diaminodiphenyl methane was studied in 3.5 wt% NaCl solution [75]. The authors concluded that the 1,6-diaminohexane-cured epoxy had higher protective efficiency than the 4,4-diaminodiphenyl-methane-cured one. Isophorone diisocyanate microcapsules, prepared by interfacial polymerization and incorporated into epoxy resin, yielded an anti-corrosive coating [76]. The coating cured on carbon steel displayed high damage healing owing to a protective polymeric barrier layer. Adding isophorone diisocyanate microcapsules and pH-sensitive cerium tri(bis(2-ethylhexyl)phosphate) particles to epoxy coating enhanced steel plate’s corrosion resistance through the combined protective effects of each component [77].

### 3.2. Epoxy Coatings Modified by Natural Organic Compounds

**Figure 8:**
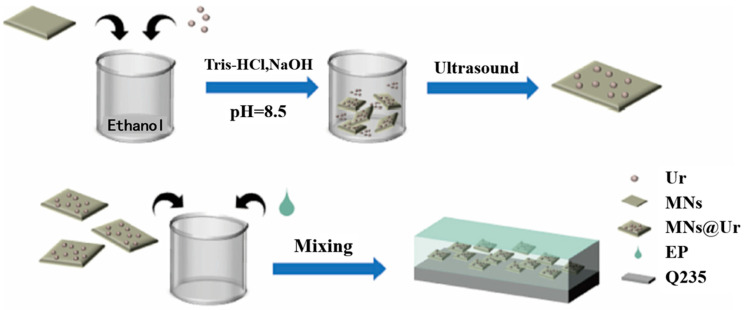
Preparation of MNs@Ur and MNs@Ur/EP composite coatings [91].

To promote the sustainability of coating materials, bio-based alternatives are of great interest for both industry and academia [78,79]. Plant oil, a widely available natural resource, has been studied as an additive or modifier in organic coatings. To develop a fully organic coating system, Ammar et al. [80] used epoxidized soybean oil to modify epoxy resin. Their research revealed that the fabricated coating system had an optimal curing level, better wettability, and good physical–mechanical properties, indicating its potential to protect the matrix from corrosion. Li et al. [81] prepared tung oil embedded in urea-formaldehyde microcapsules via in situ polymerization, which reacted with epoxy resin to form a self-healing coating. EIS tests and a salt spray experiment demonstrated that the coating had good anti-corrosion performance. Veedu et al. [82] proved that *Ixora* leaf extract could intensify the corrosion resistance of epoxy coatings, as the interaction of Fe and phytochemicals at the metal–electrolyte interface provided promising inhibition efficiency and stability against metal dissolution. They also investigated the properties of *Gliricidia sepium* leaf extract and silica hybrid [83], suggesting the -OH interacting with the metal surface promoted the corrosion protection of the epoxy matrix. Qi et al. [84] found that tannin could improve the anti-corrosion performance of Zn^2+^ and Ce^3+^. The authors identified that the synergistic effect of the co-deposited metal hydroxides/oxides and uniform iron–tannin complexes were key factors. Teijido et al. [85] synthesized epoxy coatings with various epoxide and hydroxyl groups using epoxidized soybean oil and tannic acid as natural substrates. These liquid mixtures, when painted onto a carbon steel surface, exhibited excellent anti-corrosion activity. Epoxy-Zn coatings loaded with 0.5 wt% oil palm frond cellulose nanocrystal (OPF-CNC) showed a 36% improvement in performance compared to unloaded cellulose coatings [86]. The epoxy–lignin coating was prepared using acetylated lignin for carbon steel corrosion protection [87]. Experimental data indicated that the acetylated lignin improved the modified coating’s corrosion resistance. Nunes et al. [88] synthesized a novel organic coating from low-cost cashew nut shell liquid. Compared to an uncoated steel electrode, the coated ones had a lower corrosion current density and less negative corrosion potential. Similar results on cashew nut shell liquid were reported by the Silva group [89]. Abbout et al. [90] studied the corrosion protection of carbon steel surfaces with galactomannan-modified epoxy coatings. The modified coating effectively protected the matrix in an acidic medium. Natural urushiol (Ur) was reacted with mica to prepare functional mica nanosheets (MNs) via catechol group interactions (Figure 8) [91]. The modified epoxy coating’s anti-corrosion performance improved due to the superhydrophobic properties and high dispersion of MNs@Ur. Additionally, biocide and silanized epoxy were used to synthesize a hybrid coating [92]. When applied to carbon steel, this coating demonstrated excellent anti-corrosion activity.

Natural organic compounds are renewable, biodegradable, and have a lower environmental impact compared to synthetic additives. Tannins [84], for example, are abundant in nature and can form stable complexes with metal ions, enhancing corrosion protection while reducing the need for harmful chemicals. Lignin [87] and cashew nut shell liquid [88,89] also provide sustainable alternatives for improving coating performance, aligning with the growing demand for eco-friendly materials in industrial applications.

### 3.3. Organic Coatings Modified by Other Organic Compounds

In addition to nitrogen-containing organics and natural products, other organics have also been utilized to modify epoxy resin for high-performance coatings. Zhou et al. [93] successfully synthesized acrylate copolymers via radical polymerization and incorporated these copolymer fillers into epoxy resin to prepare high-performance epoxy coatings. The results indicated that the anti-corrosion performance and self-healing characteristics of the epoxy coatings were significantly improved, attributed to the acrylate copolymer reinforcing the coating’s tightness, adhesion, hydrophobicity, and weatherability. To boost corrosion resistance in a marine environment, Chen et al. [94] developed a textured epoxy resin coating using surface texture preparation technology and reduced air bubble formation via vacuum treatment, thereby enhancing the corrosion protection of marine structural steel.

Organic-compound-modified epoxy coatings offer numerous advantages, yet their preparation methods and required time can vary significantly. Coatings modified with polyether amine D230 or those utilizing in situ polymerization of PANI typically require longer preparation times due to their complex synthesis processes. Conversely, coatings incorporating natural organic compounds, such as epoxidized-soybean-oil- or tung-oil-based microcapsules, can be prepared more quickly. These natural compounds involve simpler modification steps and more straightforward coating processes, making them more efficient in terms of preparation time.

## 4. Organometallic-Compound-Modified Epoxy Coatings

### 4.1. Epoxy Coatings Modified by Metal–Organic Frameworkds

**Figure 9:**
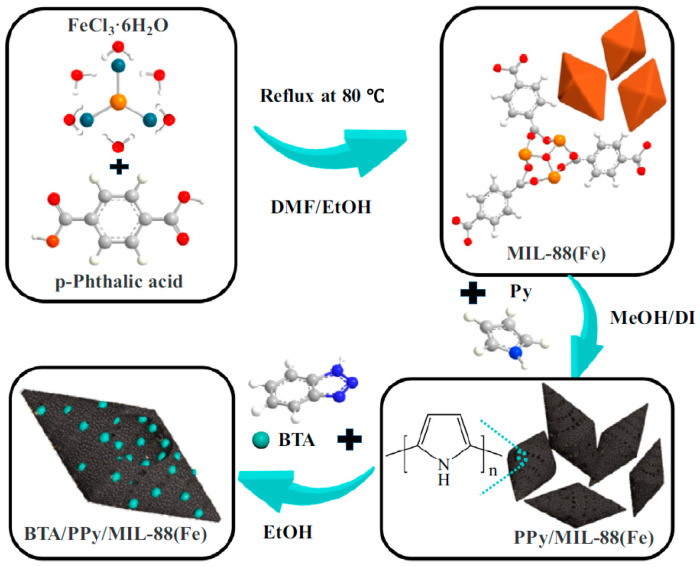
Fabrication of BTA/PPy/MIL-88(Fe) composite material [106].

Metal–organic frameworks (MOFs), a class of organometallic compounds, have broad applications in catalysis, separation, and drug delivery systems [95,96,97]. MOFs can enhance the adhesion of epoxy coatings to steel through several mechanisms. Their porous structure and high surface area allow for better interaction with the steel surface, forming strong chemical bonds. MOFs can also act as fillers, improving the mechanical interlocking between the coating and the steel. Additionally, MOFs release corrosion inhibitors that further strengthen the adhesion by forming protective layers on the steel surface. This combination of physical and chemical interactions results in enhanced coating adhesion and durability. ZIF-8 (zeolitic imidazolate framework-8) was synthesized from 2-methylimidazole and zinc nitrate tetrahydrate and then combined with graphene oxide (GO) sheets to form GO@ZIF-8 particles [98]. The results showed that adding GO improved the epoxy coating’s corrosion resistance by 70%. Xiong et al. [43] modified salicyldehyde@ZIF-8 fillers anchored on GO with 3-aminopropyltriethoxysilane. The filler improved the corrosion protection property of the epoxy coating because of its excellent dispersion stability. Zhang et al. [99] found that incorporating ZIF-8 with lubrication oil boosted the epoxy coating’s anti-corrosion performance. He et al. [100] synthesized PPy@ZIF-8 (PPy, polypyrrole) NPs via in situ polymerization and added them to epoxy resin, producing a long-term anti-corrosion coating. The long-term efficiency of the corrosion protection of the epoxy coating on steels was attributed to the self-healing behavior of the PPy@ZIF-8 particles, which facilitated the formation of a passive iron oxide and Zn (OH)_2_ deposition layer. Wan et al. [101] synthesized 5-aminotetrazole@ZIF-7@zinc gluconate/boron nitride NPs via a one-pot method using 5-aminotetrazole, zinc gluconate, benzimidazole, and boron nitride as starting materials. Owing to the synergetic effects of passive and active protection, the synthesized MOF NPs demonstrated a significant enhancement in the anti-corrosion performance of waterborne epoxy coating. Hasanzadeh and Saadatabadi [102] studied the corrosion protection properties of ZIF-67-decorated g-C_3_N_4_ nanosheets. Their findings revealed that the corrosion rate of mild steel was reduced by 50%, while its corrosion resistance was enhanced 5-fold compared to the blank sample. Zr-UIO-66 MOF, Zr-UIO-NH_2_-MOF, and Zr-NH_2_-UIO-MOF were developed and utilized as novel functional anti-corrosive fillers [103]. The test results indicated that these three Zr-MOFs could effectively improve the anti-corrosion performance of epoxy coating in 3.5% NaCl solution across various pH values (pH = 2, 7.5, and 12). Hsia et al. [104] fabricated 2D Zr-MOFs, which were incorporated into epoxy resin to boost the anti-corrosive activity of the coating. Functioning as a filler, MIL-88A (Fe) was used to modify epoxy resin so as to reinforce its anti-corrosion activity [105]. EIS and salt spray test results confirmed that the MIL-88A (Fe) particles possessed excellent barrier properties. In particular, salt spray tests showed that the adhesion loss of the epoxy coatings decreased from 31.4% to 9.4% with the addition of MIL-88A (Fe). Zhang et al. [106] developed BTA/PPy/MIL-88(Fe) through a successive oxidation and loading procedure (Figure 9). The synthesized MOFs were applied to strengthen the anti-corrosion performance of an epoxy coating. Keshmiri [107] designed and fabricated GO@Ce-MOF using GO and Ce-MOF as raw materials, after which an epoxy coating was modified by the fabricated GO@Ce-MOF filler. EIS and salt spray tests indicated that the GO@Ce-MOF/epoxy resin could enhance the anti-corrosion property of the coating. Compared to the blank sample, the anti-corrosion performance was boosted by 500%. Cu-MOFs and Zn-MOFs with unsaturated metal sites were subjected to hydrophobic modification using octadecylphosphonic acid (OPA) to prepare MOFs/OPA hydrophobic composites. The two MOFs were blended with epoxy resin to formulate hydrophobic acid-modified MOF epoxy coatings [108]. These coatings exhibited satisfactory corrosion resistance, which was attributed to the strong barrier and shielding effects. Recently, the corrosion protection properties of epoxy coating have been improved through the use of HKUST-1-BTA@e-Mica two-dimensional nanocomposites as fillers, which is credited to the enhanced mechanical properties of the coating [109].

### 4.2. Epoxy Coatings Modified by Other Organometallic Compounds

**Figure 10:**
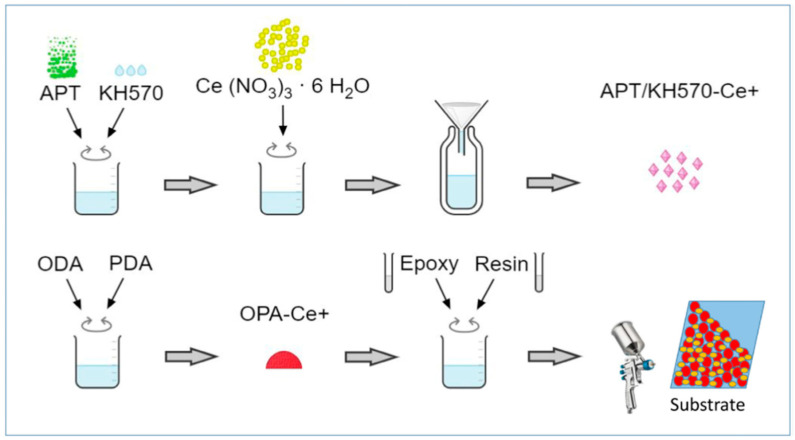
Preparation of OPA–Ce^3+^ epoxy coating [111].

To enhance the corrosion resistance of coatings, other organometallic compounds have also been employed as fillers to modify epoxy coatings. Attaei et al. [77] modified epoxy resin with isophorone diisocyanate and pH-sensitive cerium tri(bis(2-ethylhexyl)phosphate, and the resulting coating exhibited the dual functions of self-healing and corrosion resistance. Cu(II) and Zr(IV) complexed with metformin and 2,2-bipyridine were incorporated into epoxy resin to formulate multi-functional coatings [110]. Salt spray and acid spot tests revealed that the obtained coatings, particularly the modified Cu(II) epoxy coating, offered excellent corrosion protection properties. Carbon steel was treated via a pulse-reverse electrodeposition method in a bath containing 3-nitrobenzoic acid and zinc nitrate, after which the treated carbon steel was coated with epoxy resin [17]. The experimental results indicated that the adhesion strength and anti-corrosion performance of the epoxy coating on the treated matrix were improved. Given the strong chelation ability of Ce^3+^ with organics, Butt et al. [111] designed and synthesized ODA/PDA/APT-Ce^3+^ (OPA–Ce^3+^) coatings using octadecylamine (ODA) and cerium nitrate as raw materials (Figure 10). The synthesized coating exhibited superhydrophobic, self-healing, and self-cleaning properties, suggesting its potential for effectively protecting stainless steel from corrosion. Chopra et al. [112] investigated the corrosion inhibitive effect of ZA (acetoxime derivative of zinc chloride). They found that the epoxy coating modified with ZA possessed excellent barrier characteristics due to the formation of Zn–O–C and O–Zn–O linkages. Peng et al. [113] studied the impact of lanthanum 4-hydroxycinnamate and 3-(4-methylbenzoyl)propanoate on the corrosion resistance of epoxy coatings. EIS analysis evidenced that the rare earth carboxylate could offer anti-corrosion inhibition for steel. Similarly, Zhang et al. [114] demonstrated that rare earth carboxylate-containing coatings exhibited outstanding corrosion protection properties. To address the low anti-corrosion efficacy of inorganic cerium salts for carbon steel, Ren et al. [115] developed an organic cerium phosphate complex as a rapid repair agent. The corrosion resistance of the formed film on Q235 steel was enhanced by the repair capabilities of the organic cerium complex.

## 5. Carbon-Material-Modified Epoxy Coatings

### 5.1. Graphene-Modified Epoxy Coatings

**Figure 11:**
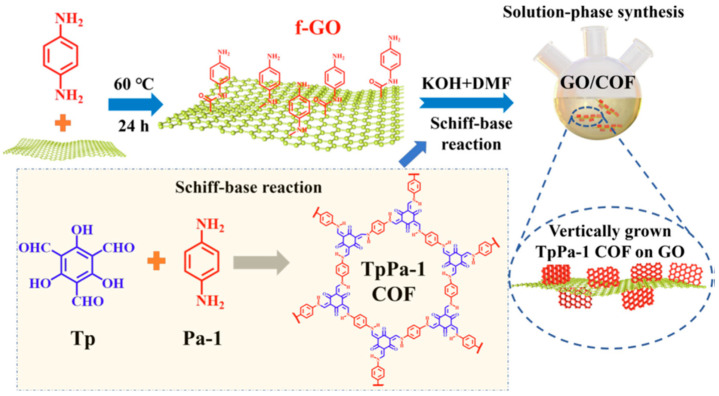
Synthesis of GO/COF [117].

Recently, the application of graphene and graphene oxides (GOs) in the modification of epoxy coatings has garnered significant attention [116,117,118,119]. Yan et al. [119] fabricated an ideal Ti_3_C_2_/graphene hybrid using Ti_3_AlC_2_ and graphene as raw materials. The graphene could decrease the microstructural defects and reinforce the adhesion of the epoxy coating [120]. Compared to the pure epoxy coating, the graphene-modified epoxy coating had higher anti-corrosion capacity [120]. As an additive, the Ti_3_C_2_/graphene hybrid enhanced the anti-corrosive properties of the organic coating because of its synergistic effect and thermal conductivity. Ye et al. [121] verified that a coating modified with graphene had excellent self-healing capacity due to the spontaneous release from a graphene-based nanocontainer. Madhusudhana et al. [116] investigated the anti-corrosion ability of functionalized graphene oxide–epoxy coating on mild steel in 3.5 wt% NaCl solution. An epoxy phenolic novolac polymer was modified by GO, which was fabricated by refluxing a mixture of epoxy and phenol formaldehyde amine. The results indicated that a corrosion inhibition efficiency of 99.99% was achieved with the 0.2 wt% functionalized GO-grafted epoxy phenolic novolac coating. Ramezanzadeh et al. [98] developed ZIF-8-decorated GO to enhance the anti-corrosion performance of an epoxy coating. Compared to the blank sample, the corrosion resistance of the coating modified with GO@ZIF-8 was improved by 70%. Silanized Cr_3_C_2_ was encapsulated with GO and incorporated as a nanofiller into an epoxy coating [122]. EIS testing revealed that the modified epoxy coating exhibited significantly higher corrosion resistance compared to the unmodified epoxy coating. Organic-functionalized GOs have been extensively investigated due to their remarkable corrosion protection properties [123,124]. To further enhance the anti-corrosion performance, GO was functionalized with phytic acid and subsequently incorporated into a water-borne epoxy coating [125]. Electrochemical measurements and salt spray tests implied that the corrosion resistance was remarkable, which was primarily attributed to the excellent dispersity of the functionalized GO within the coating matrix. Zhu et al. [126] explored the anti-corrosion performance of polypyrrole-functionalized GO synthesized via an in situ polymerization method. EIS analysis indicated that the functionalized GO (at a concentration of 0.05%) coating exhibited significant corrosion protection performance. GO was functionalized using 3-amino-1, 2, 4-triazole-5 thiol and was used to enhance the corrosion protection performance of an epoxy coating on mild steel [124]. Compared to the neat epoxy coating, the modified matrix exhibited higher film resistance, charge transfer resistance, and low capacitance. GO functionalized with 1-butyl-3-methylimidazolium acetate was uniformly dispersed within an epoxy coating, thereby strengthening the anti-corrosive properties of the modified epoxy coating on carbon steel [123]. Electrochemical studies evidenced that the modified coating achieved a higher corrosion protection efficiency (about 99%) in 3.5 wt% NaCl solution compared to the blank sample. Xiang et al. [127] synthesized GO grafted with 4-amino-antipyrine (AAP) via a hydrothermal method. All data suggested that the synergistic effect of physical shielding and corrosion inhibition imparted by GO@AAP could significantly enhance the anti-corrosion performance of waterborne epoxy coating on Q235 mild steel in a 3.5 wt% NaCl solution. Li et al. [128] employed amino-modified graphene oxide, synthesized using urea and melamine, to bolster the corrosion protection of epoxy coatings on a Q235 steel surface. Diraki and Omanovic [129] fabricated a double-layer PANI-GO/epoxy coating to protect carbon steel in a saltwater environment. Specifically, conductive PANI loaded with GO was initially coated onto the carbon steel, followed by coverage with a commercial epoxy coating to construct the double-layer structure. The experimental results demonstrated that the fabricated coating possessed high anti-corrosion resistance, as the PANI-GO layer effectively hindered the transport of hydrated corrosive ions to the carbon steel surface. To address the issues of poor dispersion and high mechanical damage associated with GO, Zhang et al. [117] synthesized covalent organic framework (COF)-modified GO to further elevate the anti-corrosion performance of epoxy coatings (Figure 11). Subsequently, the synthesized GO/COF was loaded with benzotriazole (BTA) to produce GO/COF-BTA filler. EIS testing revealed that the epoxy coating reinforced with GO/COF@BTA-2% exhibited superior corrosion protection and self-healing ability, attributed to its good compatibility and higher crosslinking density and the controllable release of BTA. The application of reduced GO (rGO) was also explored to enhance the anti-corrosion performance of epoxy coatings [118,130]. Kooshksara and Mohammadi [130] treated multi-layered GO using an in situ solvothermal reduction method to obtain rGO. Long-term salt spray and EIS studies confirmed that rGO enhanced the corrosion protection of epoxy coatings owing to its high-quality dispersion. Additionally, rGO functionalized with PANI and was employed to improve the corrosion protection of epoxy coatings [118]. The authors revealed that the high surface area and remarkable structural barrier of the functionalized rGO provided excellent protective coverage in a 3.5 wt% NaCl solution. Moreover, graphene composited with metal oxide or MOFs has been applied to enhance the anti-corrosion performance of epoxy coatings [6,57,131].

### 5.2. Epoxy Coatings Modified by Other Carbon Materials

**Figure 12:**
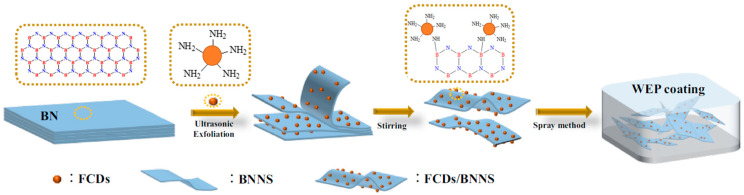
Preparation of waterborne epoxy coating modified by amino-functionalized carbon dots\boron nitride nanosheets [136].

Except for graphene and graphene oxide, some other carbon martials have been explored to enhance the corrosion protection of epoxy coatings. Chen et al. [132], for the first time, utilized g-C_3_N_4_ to disperse graphene in an epoxy solution via an ultrasonic vibration method. EIS and salt spray tests revealed that the modified epoxy coating exhibited enhanced anti-corrosion performance, primarily attributed to the strengthened barrier properties of g-C_3_N_4_ and graphene. Recently, Hasanzadeh and SaadatAbadi [102] found that ZIF-67-decorated g-C_3_N_4_ nanosheets could significantly improve the self-healing capacity and corrosion resistance of epoxy coatings. Graphitic carbon nitride was employed to encapsulate MnO_2_ modified by benzidine within pure epoxy resin, and the resulting decorated epoxy coating demonstrated high corrosion protection efficacy owing to the increased adhesive strength [133]. As an anti-corrosive additive, PNAI/CNT (CNT, carbon nanotube) hybrids were functionalized with 1-methyl-3-butyl imidazolium tetrafluorate [134]. The findings suggested that the epoxy coating loaded with 1 wt% of the synthesized PANI/CNT hybrid presented outstanding anti-corrosion properties. An epoxy coating was modified with functionalized multi-walled carbon nanotube/polyindole fillers and tested on mild steel in a 3.5 wt.% NaCl solution [135]. The investigations indicated that the coating containing 0.25 wt% fillers exhibited excellent anti-corrosion and barrier protection properties. Furthermore, boron nitride modified by amino-functionalized carbon dots was also incorporated into waterborne epoxy resin to enhance its anti-corrosive properties (Figure 12) [136]. The synergistic effects of these composites markedly bolstered the anti-corrosive ability of the epoxy coating.

When considering the large-scale introduction of new carbon-material-modified epoxy coatings, multiple indicators suggest a favorable trajectory. For instance, materials such as g-C_3_N_4_ and PANI/CNT hybrids have demonstrated significant potential in improving corrosion resistance. Moreover, the development of boron nitride modified with amino-functionalized carbon dots presents a promising avenue for enhancing barrier properties. However, it is crucial to acknowledge that challenges like cost-effective synthesis and consistent industrial-scale performance must be overcome. In summary, while certain materials are nearing commercial viability, others remain in research phases, signaling a gradual yet steady upward trend in their large-scale application.

## 6. Conclusions and Outlook

As promising corrosion-resistant materials, organic epoxy coatings modified by various additives will be researched in-depth and applied widely in industry in the future. Our review highlights the diversity and innovation in modifying epoxy coatings with different categories of additives, including metals and their compounds, organic compounds, organometallic compounds, and carbon materials. Each category offers unique advantages and addresses specific corrosion challenges, expanding the scope of protective coating solutions.

Zinc-rich organic epoxy coatings, when modified with organic compounds or metal oxides, continue to be industrially attractive due to their robust performance and established application basis. Among the various modifiers, nitrogen-containing organic compounds, such as PANI, stand out because they form M-N bonds with metal surfaces, enhancing coating adhesion and preventing steel corrosion. The hybrid use of organics and metal oxides not only blocks corrosive agents but also boosts coating corrosion resistance through M-O-Fe or M-N-Fe bond formation, a strategy that has garnered significant research interest.

Metal–organic compounds, particularly MOFs, have shown great potential in reinforcing the anti-corrosive activity of epoxy coatings. Their structural tunability and compatibility with epoxy resins make them promising candidates for developing high-performance modified coatings. Additionally, natural materials, with their wide range of sources and dual organic–inorganic properties, offer sustainable alternatives for enhancing the anti-corrosion properties of epoxy coatings, though further research is needed to identify the key active components.

Carbon materials, including graphene and graphene oxides, will continue to be thoroughly researched as fillers for their ability to hybridize with other substances and improve coating performance.

While composite epoxy resin coatings offer improved performance, they also face challenges such as dispersion difficulties and cost considerations. Some additives, especially nanomaterials, tend to aggregate, leading to uneven distribution and reduced effectiveness. Proper dispersion techniques and surface modifications are essential to address this issue. Cost-effectiveness is another concern, as some advanced additives and modification methods can increase production costs. Balancing performance improvements with economic viability is crucial for the widespread adoption of these coatings in industrial applications.

Future research in modified organic epoxy resin coatings for steel could explore several promising directions. The development of more efficient and cost-effective dispersion techniques would enhance the performance of these coatings. Investigating the long-term durability and environmental impact of composite coatings is also essential. Additionally, the integration of smart materials and self-healing technologies could further improve corrosion protection. Exploring new additive combinations and hybrid systems may lead to innovative solutions for specific industrial challenges. Finally, advancing the understanding of the fundamental mechanisms behind the improved properties of composite coatings will guide the design of next-generation materials.

Composite epoxy resin coatings modified with various additives show great potential for enhancing steel corrosion resistance in diverse industrial environments. This review highlights the diversity and innovation in modifying epoxy coatings with different categories of additives. Zinc-rich coatings modified with organic compounds or metal oxides continue to be industrially attractive. The hybrid use of organics and metal oxides not only blocks corrosive agents but also boosts coating corrosion resistance through bond formation, a strategy that has garnered significant research interest. Metal–organic compounds, particularly MOFs, have shown great potential in reinforcing the anti-corrosive activity of epoxy coatings. Natural materials offer sustainable alternatives for enhancing the anti-corrosion properties of epoxy coatings, though further research is needed to identify key active components. Carbon materials, including graphene and graphene oxides, will continue to be thoroughly researched as fillers for their ability to hybridize with other substances and improve coating performance. Future research should focus on developing more high-performance modified epoxy resins, leveraging the unique properties of these diverse additives to enhance steel corrosion resistance in various industrial environments.

## Figures and Tables

**Table 1:** Summary of benefits and limitations of various corrosion protection methods involving metals and their compounds.

Corrosion Protection Method	Benefits	Limitations
Epoxy coatings modified by metals and their compounds	- Significantly enhance corrosion resistance - Improve barrier properties - Provide active corrosion protection	- Some metal compounds may have limited dispersibility - Potential for micro-crack formation - Cost considerations for certain metals
Zinc-rich epoxy coatings	- Excellent resistance to chemical corrosion - Good cathodic protection for carbon steel - Effective under highly acidic media	- Require specific application techniques - Cost of zinc particles - Environmental concerns with zinc disposal
Titanium-modified epoxy coatings	- Enhanced corrosion protection in harsh environments - Improved thermal and UV resistance - Effective barrier against corrosive species	- Higher material costs - Complex processing requirements - Potential brittleness issues
Silicon-modified epoxy coatings	- Superior mechanical and thermal properties - Enhanced adhesion and corrosion resistance - Effective under high-temperature conditions	- Silane modification requires precise control - Limited availability of certain silicon compounds - Cost of specialized silicon additives
Epoxy coatings modified by other metal compounds	- Low cost and easy synthesis - Chemical stability - Synergistic effects with other additives	- Potential environmental and health concerns - Limited solubility in some coating systems - Performance depends on particle size and distribution

## Data Availability

No new data were created or analyzed in this study.
